# THC Prevents MDMA Neurotoxicity in Mice

**DOI:** 10.1371/journal.pone.0009143

**Published:** 2010-02-10

**Authors:** Clara Touriño, Andreas Zimmer, Olga Valverde

**Affiliations:** 1 Departament de Ciències Experimentals i de la Salut, Grup de Recerca en Neurobiologia del Comportament (GRNC), Universitat Pompeu Fabra, Barcelona, Spain; 2 Department of Molecular Psychiatry, University of Bonn, Bonn, Germany; Minnesota State University Mankato, United States of America

## Abstract

The majority of MDMA (ecstasy) recreational users also consume cannabis. Despite the rewarding effects that both drugs have, they induce several opposite pharmacological responses. MDMA causes hyperthermia, oxidative stress and neuronal damage, especially at warm ambient temperature. However, THC, the main psychoactive compound of cannabis, produces hypothermic, anti-inflammatory and antioxidant effects. Therefore, THC may have a neuroprotective effect against MDMA-induced neurotoxicity. Mice receiving a neurotoxic regimen of MDMA (20 mg/kg ×4) were pretreated with THC (3 mg/kg ×4) at room (21°C) and at warm (26°C) temperature, and body temperature, striatal glial activation and DA terminal loss were assessed. To find out the mechanisms by which THC may prevent MDMA hyperthermia and neurotoxicity, the same procedure was carried out in animals pretreated with the CB_1_ receptor antagonist AM251 and the CB_2_ receptor antagonist AM630, as well as in CB_1_, CB_2_ and CB_1_/CB_2_ deficient mice. THC prevented MDMA-induced-hyperthermia and glial activation in animals housed at both room and warm temperature. Surprisingly, MDMA-induced DA terminal loss was only observed in animals housed at warm but not at room temperature, and this neurotoxic effect was reversed by THC administration. However, THC did not prevent MDMA-induced hyperthermia, glial activation, and DA terminal loss in animals treated with the CB_1_ receptor antagonist AM251, neither in CB_1_ and CB_1_/CB_2_ knockout mice. On the other hand, THC prevented MDMA-induced hyperthermia and DA terminal loss, but only partially suppressed glial activation in animals treated with the CB_2_ cannabinoid antagonist and in CB_2_ knockout animals. Our results indicate that THC protects against MDMA neurotoxicity, and suggest that these neuroprotective actions are primarily mediated by the reduction of hyperthermia through the activation of CB_1_ receptor, although CB_2_ receptors may also contribute to attenuate neuroinflammation in this process.

## Introduction

3,4-Methylenedioxymethamphetamine (MDMA), commonly known as ecstasy, is a widely used recreational drug with low addictive potential, but with severe neurotoxic effects after prolonged use [1]. MDMA produces the loss of 5-HT nerve terminals when administered to primates or rats [2], [3], and the degeneration of dopamine (DA) nerve terminals when administered to mice [4]. MDMA also induces hyperthermia [5], which enhances neurotoxicity. In addition, MDMA-induced hyperthermia increases at warm ambient temperature, what, consequently, aggravates axonal degeneration. High temperature enhances the formation and uptake of MDMA toxic metabolites that increase oxidative stress [6], causing nerve terminal damage [7], [8], and leading to neuroinflammation manifested by glial activation [9], [10], and eventually axonal degeneration. The strong influence of ambient temperature on MDMA neurotoxicity is of clinical interest since MDMA is frequently consumed in dance clubs with warm ambient temperatures [11], where the neurotoxic effects of the drugs may be exacerbated. One of the most effective mechanisms to minimize MDMA neurotoxicity is to reduce hyperthermia by decreasing ambient temperature [12] or using antithermic drugs to control body temperature [9]. However, these drugs are rarely consumed by MDMA users. Interestingly, one of the drugs most frequently consumed together with MDMA is cannabis [13], [14]. Δ^9^-tetrahydrocannabinol (THC), the main psychoactive compound of cannabis, has widely reported hypothermic [15], anti-inflammatory [16] and antioxidant [17] properties. Indeed, MDMA and THC show many opposite pharmacological effects. MDMA causes hyperlocomotion, hyperthermia, anxiety, and neurotoxicity [5], whereas THC induces hypolocomotion, hypothermia, anxiolytic, and neuroprotective properties [15]. Several studies in animal models demonstrate that the combination of MDMA and THC counterbalances many of their pharmacological effects. THC attenuated MDMA-induced hyperlocomotion, hyperthermia, and anxiety in rats [18], and MDMA reduced THC withdrawal syndrome in mice [19]. However, the neuroprotective effects of THC on MDMA neurotoxicity have not yet been explored. The opposite effects of cannabis and MDMA suggest that THC may provide some degree of protection against the neurotoxic effects of MDMA [20], [21]. Therefore, in the present study, we investigate if hypothermic and neuroprotective properties of THC may prevent the neurotoxic effects of MDMA in mice.

## Materials and Methods

### Animals

We used 9 to 12 week old male C57BL/6 mice for this study. Mice were either wild-type (Charles River, France) or deficient in the CB_1_ and/or CB_2_ cannabinoid receptors [22], [23]. All animals were housed in a temperature (21° or 26°±1°C), humidity (55%±10%), and light-cycle controlled room.

Food and water were available *ad libitum*. Light was on between 8:00 am and 8:00 pm, and the experiments took place during the light phase. All animal care and experimental procedures were conducted according to the guidelines of the European Communities Directive 86/609/EEC regulating animal research and were approved by the local ethical committee (CEEA-PRBB).

### Drugs

MDMA hydrochloride (Lipomed, A.G., Arlesheim, Switzerland) was dissolved in 0.9% saline and administered at 20 mg/kg, i.p. four times every 2 h. THC (THC Pharm, Frankfurt, Germany) was dissolved in a solution of 5% ethanol, 5% cremophor EL (Sigma Chemical, Madrid, Spain) and 90% physiological saline (0.9%), and administered at 3 mg/kg, i.p. 1 h before each MDMA injection. The CB_1_ receptor antagonist AM251 and the CB_2_ receptor antagonist AM630 (Tocris Bioscience, Bristol, UK) were dissolved in a solution of 5% DMSO, 5% Tween 80 (Sigma-Aldrich, Madrid Spain) and 90% physiological saline, and administered at 1 mg/kg, i.p. 15 min before each THC injection. All these drugs were administered in a volume of 0.1 ml/10 g. Ketamine hydrochloride (100 mg/kg; Imalgène 1000®, Rhône Mérieux, Lyon, France) and xylazine hydrochloride (20 mg/kg; Sigma Chemical Co., Madrid, Spain) were mixed and dissolved in ethanol and water (1∶9). This anesthetic mixture was injected in a volume of 0.2 ml/10 g body weight i.p., and used for intracardiac perfusion.

### Experimental Procedure

Animals were injected with MDMA (20 mg/kg, i.p.) or saline every 2 h for a total of 4 injections. One hour before each MDMA administration, animals received an injection of THC (3 mg/kg, i.p.) or its corresponding vehicle. A group of animals were also pretreated with the CB_1_ receptor antagonist AM 251 or the CB_2_ receptor antagonist AM 630 15 min before each THC injection. Body temperature was determined 30 min after the first MDMA injection. 48 h after the last MDMA injection, animals were sacrificed for immunostaining or western blot analysis.

### Body Temperature

Rectal temperature was measured in animals housed at 21 and at 26±1°C and treated with THC and MDMA or their corresponding vehicles. Body temperature was measured by placing an electronic thermocouple flexible rectal probe (Panlab, Madrid, Spain) in the rectum for 10 s. Temperature was measured 30 min after the first MDMA injection. In order to determine the mechanism by which THC prevents MDMA-induced hyperthermia, two additional experiments were performed; (1) temperature was recorded in animals housed at 26±1°C and treated with the CB_1_ receptor antagonist AM251 or the CB_2_ receptor antagonist AM630, at 1 mg/kg 15 min before THC injection, and (2) body temperature was measured in CB_1_, CB_2_ and double CB_1_/CB_2_ knockout mice housed 26±1°C and treated with THC and MDMA.

### Immunostaining

Activated microglia and astrocytes, and DA axons were identified by immunohistochemistry in the striatum of animals housed at 21 and 26±1°C and treated with MDMA and THC. Mice were anesthetized 48 h after the last MDMA injection with a ketamine/xylazine mixture and transcardially perfused with 0.1 M phosphate buffer containing 4% paraformaldehyde. Brains were removed and postfixed in the same solution for four hours and cryoprotected in 30% sucrose overnight. After freezing in dry ice, brains were sliced into 30-µm thick coronal sections containing the striatum. Sections were preincubated for 30 min in 20% H_2_O_2_ (Sigma-Aldrich, Spain), and then incubated for 2 h in a solution of 3% normal goat serum (Vector Laboratories, Inc., Burlingame, CA) and 0.3% triton X-100 (Sigma-Aldrich, Spain). Activated microglia was detected with rat anti-mouse CD11b (1∶100; Serotec, Oxford, UK), astrocytes were detected with polyclonal rabbit anti-glial fibrillary acidic protein (1∶1000; GFAP) (Dako, Glostrup, Denmark), and DA axons were detected with mouse anti-TH (1: 5000; Sigma-Aldrich, Spain). To visualize anti-CD11b and anti-TH primary antibodies, biotinylated secondary antibodies to rat or mouse Igs were applied for 1 h, followed by incubation with avidin/biotin reagent (Vector Laboratories, Inc., Burlingame, CA) for 2 h. Sections were stained with diaminobenzidine-HCl (DAB) and H_2_O_2_. A fluorescent Alexa Fluor® 488 secondary antibody (Invitrogen, Eugene, OR) was used to visualize anti-GFAP primary antibody. Analysis of CD11b and GFAP immunostaining in the striatal region was carried out with Image J software [24]. Briefly, the area comprising the striatum was selected and the background was subtracted by adjusting detection threshold density considering just the signal density above the threshold. The number of pixels per area was measured automatically, and the percentage of stained area was determined. Measurements were performed in 3 different slices from the same animal, the average was calculated, and the data were expressed as percentage of the stained area.

### Western Blot Analysis

Analysis of protein levels of tyrosine hydroxylase (TH), tryptophan hydroxylase (TrH), serotonin transporter (SERT), and glyceraldehyde-3-phosphate dehydrogenase (GAPDH) in the striatum, as well as the levels of TrH and SERT in prefrontal cortex and hippocampus of animals housed at 21 or 26±1°C and treated with THC and MDMA were analyzed by western blot. Animals were sacrificed 48 h after the last MDMA injection, and the striatum was dissected. Samples from all animals were processed in parallel to minimize inter-assay variations. Frozen brain areas were dounce-homogenized in 30 volumes of lysis buffer (50 mmol/L Tris-HCl pH 7.4, 150 mmol/L NaCl, 10% glycerol, 1 mmol/L EDTA, 1 µg/mL aprotinin, 1 µg/mL leupeptine, 1 µg/mL pepstatin) plus 1% Triton X-100. After 10 min incubation at 4°C, samples were centrifuged at 16000 g for 30 min to remove insoluble debris. Supernatant protein contents were determined by DC-micro plate assay (Bio-Rad, Madrid, Spain), following the manufacturer's instructions.

Equal amounts of brain lysates were mixed with denaturing 5x Laemmli loading buffer and boiled for 5 min at 95°C. Samples with equal amounts of total protein (20 µg per lane) were separated in 10% sodium dodecyl sulfate-polyacrylamide gel before electrophoretic transfer onto immobilon membrane (Millipore, Billerica MA). Membranes were blocked for 1 h at room temperature in Tris-buffered saline (TBS) (100 mmol/L NaCl, 10 mmol/L Tris, pH 7.4) with 0.1% Tween-20 (TBS-T) and 5% non-fat milk. Afterwards, membranes were incubated for 2 h with mouse anti-TH (1∶5000; Sigma-Aldrich, Spain), rabbit anti-SERT (1∶1000), sheep anti TrH (1∶1000) (Millipore, Billerica MA), and mouse anti-GAPDH (1∶5000) (Santa Cruz Biotechnology, Santa Cruz, CA) primary antibodies. Bound antibodies were detected with horseradish peroxidase-conjugated and anti-mouse, anti-rabbit (Pierce, Spain; diluted), and anti-sheep (1∶2500; Santa Cruz Biotechnology, Santa Cruz, CA) secondary antibodies and visualized by enhanced chemiluminescence detection (SuperSignal, Pierce, Spain). Only immunoblots showing similar amount of GAPDH in all lanes were considered. The relevant immunoreactive bands were quantified after acquisition on a Chemiluminescent Imaging with Chemi-Doc XRS, controlled by Image Gauge software (Fuji, Japan). Data was expressed as the percentage of the band intensity compared to the control.

### Statistical Analysis

Differences in body temperature, and microglia and astrocytes staining between the different genotypes were compared by two-way ANOVA with treatment and genotype as between-subjects factors of variation, followed by one-way ANOVA and subsequent *post hoc* analysis (Tukey's test). Differences in body temperature and glial staining between animals housed at different ambient temperatures were compared by two-way ANOVA with treatment and ambient temperature as between-subjects factors of variation, followed by one-way ANOVA (Table 1 and 2). Differences in body temperature of animals housed at 21°C, and microglia and astrocytes staining, TH, TrH and SERT protein levels of animals housed at 26°C were compared by one-way ANOVA followed by *post hoc* analysis (Tukey's test). In all the experiments, differences were considered significant if the probability of error was less than 5%.

**Table 1:** One-way ANOVA calculated for body temperature, CD11b and GFAP staining and TH levels at 21 and 26°C.

	Factor		F-value	p-value
**Body temperature (21°C)**	**Treatment**		F_(1, 39)_ = 20.595	<0.001
**CD11b (21°C)**	**Genotype**	vehicle	F_(3, 21)_ = 0.959	n.s.
		MDMA	F_(3, 27)_ = 0.449	n.s.
		THC+MDMA	F_(3, 15)_ = 49.549	<0.001
	**Treatment**	WT	F_(2,26)_ = 38.656	<0.001
		CB_1_ KO	F_(2, 12)_ = 25.853	<0.001
		CB_2_ KO	F_(2, 12)_ = 32.976	<0.001
		CB_1_-CB_2_ KO	F_(2, 13)_ = 55.084	<0.001
**GFAP (21°C)**	**Genotype**	vehicle	F_(3, 19)_ = 0.959	n.s.
		MDMA	F_(3, 26)_ = 1.028	n.s.
		THC+MDMA	F_(3, 14)_ = 17.375	<0.001
	**Treatment**	WT	F_(2, 21)_ = 51.671	<0.001
		CB_1_ KO	F_(2, 13)_ = 23.213	<0.001
		CB_2_ KO	F_(2, 13)_ = 25.361	<0.001
		CB_1_-CB_2_ KO	F_(11, 187)_ = 121.025	<0.01
**TH (21°C)**	**Treatment**		F_(2, 9)_ = 4.434	n.s.
**Body temperature (26°C)**	**Genotype**	vehicle	F_(3, 28)_ = 0.678	n.s.
		THC	F_(3, 28)_ = 5.006	<0.01
		MDMA	F_(3, 35)_ = 1.026	n.s.
		THC+MDMA	F_(3, 32)_ = 7.010	<0.001
	**Treatment**	WT	F_(3, 32)_ = 23.044	<0.001
		CB_1_ KO	F_(3, 29)_ = 11.080	<0.001
		CB_2_ KO	F_(3, 37)_ = 16.899	<0.001
		CB_1_-CB_2_ KO	F_(3, 22)_ = 42.854	<0.001
**AM251**	**Treatment**		F_(5, 39)_ = 18.028	<0.001
**AM630**	**Treatment**		F_(5, 43)_ = 14.583	<0.001
**Body temperature (21 vs 26°C)**	**Ambient temperature**	vehicle	F_(1, 22)_ = 1.519	n.s.
		THC	F_(1, 20)_ = 0.019	n.s.
		MDMA	F_(1, 22)_ = 16.831	<0.001
		THC+MDMA	F_(1, 24)_ = 12.906	<0.01
**CD11b (26°C)**	**Treatment**		F_(2, 9)_ = 61.087	<0.001
**GFAP (26°C)**	**Treatment**		F_(2, 9)_ = 60.350	<0.001
**TH levels (26°C)**	**Treatment**	WT	F_(2, 9)_ = 72.558	<0.001
		CB_1_ KO	F_(2, 9)_ = 80.098	<0.001
		CB_2_ KO	F_(2, 9)_ = 151.349	<0.001
		CB_1_-CB_2_ KO	F_(2, 9)_ = 30.274	<0.001
**AM251**	**Treatment**		F_(3, 12)_ = 30.274	<0.001
**AM630**	**Treatment**		F_(3, 12)_ = 43.394	<0.001

One-way ANOVA for genotype, treatment or ambient temperature as between-subject factors. See Materials and methods for details. WT: wild-type; KO: knockout; n.s.: non-significant.

**Table 2:** Two-way ANOVA calculated for body temperature and astrocytes and microglia activation in mice treated with THC and MDMA.

**21°C**		**Genotype**	**p-value**	**Treatment**	**p-value**	**Interaction**	**p-value**
**Body temperature**	**Genotype x treatment**	F_(3, 120)_ = 10.007	P<0.001	F_(3, 120)_ = 44.483	p<0.001	F_(9, 120)_ = 4.540	p<0.001
**CD11b**	**Genotype x treatment**	F_(3, 63)_ = 6.673	p<0.001	F_(2, 63)_ = 87.306	p<0.001	F_(6, 63)_ = 6.921	p<0.001
**GFAP**	**Genotype x treatment**	F_(3, 59)_ = 6.491	p<0.001	F_(2, 59)_ = 85.306	p<0.001	F_(6, 59)_ = 4.120	p<0.01
**26°C**		**Ambient temperature**		**Treatment**		**Interaction**	
**Body temperature**	**Ambient temperature x treatment**	F_(1, 88)_ = 18.498	p<0.001	F_(3, 88)_ = 35.861	p<0.001	F_(3, 88)_ = 5.270	p<0.001
**CD11b**	**Ambient temperature x treatment**	F_(1, 29)_ = 6.417,	p<0.05	F_(2, 29)_ = 47.921	p<0.001	F_(2, 29)_ = 6.725	p<0.01
**GFAP**	**Ambient temperature x treatment**	F_(1, 26)_ = 16.683	p<0.001	F_(2, 26)_ = 107.930	p<0.01	F_(2, 26)_ = 16.744	p<0.001

Two-way ANOVA with genotype, ambient temperature or treatment as between-subject factors. See Materials and methods for details.

## Results

### Effects of THC on MDMA Treatment at Room Temperature (21±1°C)

#### THC reverses the moderated hyperthermia induced by MDMA at room temperature

**Figure 1:**
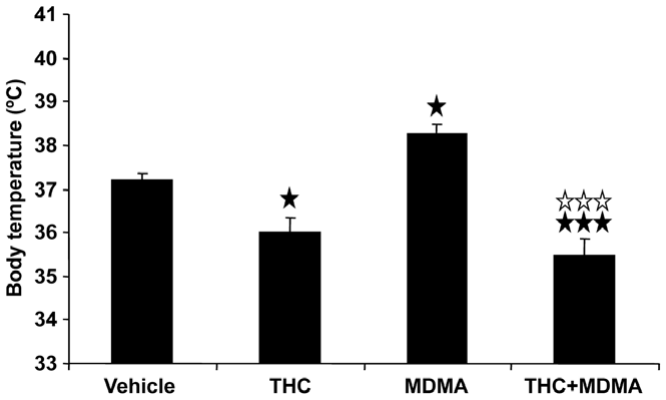
THC prevents MDMA-induced hyperthermia at room temperature. Effects of THC (3 mg/kg, i.p.) on MDMA (20 mg/kg, i.p.)-induced hyperthermia in animals housed at 21±1°C. Body temperature was measured 30 min after each the first MDMA injection. Data are expressed as mean ± SEM of the body temperature increase (*n* = 10). ★ *p*<0.05, ★★★ *p*<0.001 when compared with vehicle-treated group. ⋆⋆⋆ *p*<0.001, when compared with MDMA-treated group (Tukey's test).

A baseline body temperature of 37.5±0.08°C was recorded in all animals housed at 21±1°C. One hour after baseline, animals were injected with THC or vehicle followed by MDMA or saline. Body temperature was measured 30 min after MDMA injection (Fig. 1). Animals treated with MDMA showed a statistically significant increase in body temperature compared to the vehicle-treated group (p<0.05). In contrast, a reduction of body temperature was observed in animals treated with THC (p<0.05). Likewise, THC significantly reduced body temperature in MDMA-treated animals when compared to MDMA-treated mice (p<0.001), and when compared with the saline-treated group (p<0.001) (Table 1).

#### THC prevents microglia and astrocytes activation in MDMA-treated mice, mainly by CB1 but also by CB2 receptor-mediated mechanism

**Figure 2:**
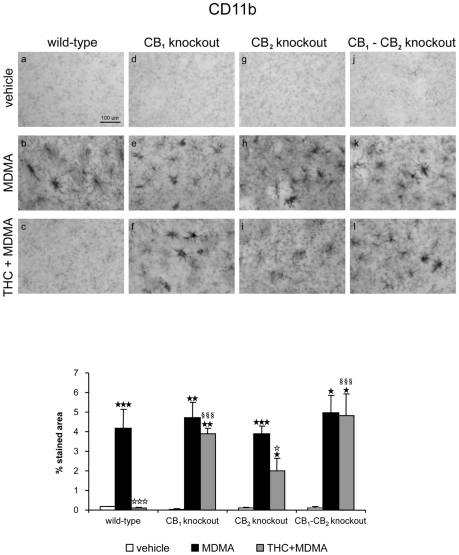
THC prevents MDMA-induced microglial activation through CB_1_ and CB_2_ receptor activation. Upper panel: CD11b staining in the striatum of wild-type (a–c), CB_1_ (d–f) CB_2_ (g–i), and double CB_1_/CB_2_ (j–l) knockout mice treated with MDMA (20 mg/kg, i.p.) and THC (3 mg/kg, i.p.), and housed at 21±1°C Animals were sacrificed and brains removed 48 h after the last injection. Scale bar, 100 µm. Lower panel: Data on microglial staining quantification (lower panel) are expressed as mean ± SEM of percentage of stained area (*n* = 3–6). ★ *p*<0.05, ★★ *p*<0.01, ★★★ *p*<0.001 when compared with vehicle-treated group. ⋆ *p*<0.05, ⋆⋆⋆ *p*<0.001, when compared with MDMA-treated group. §§§ *p*<0.001, when compared with wild-type group (Tukey's test).

**Figure 3:**
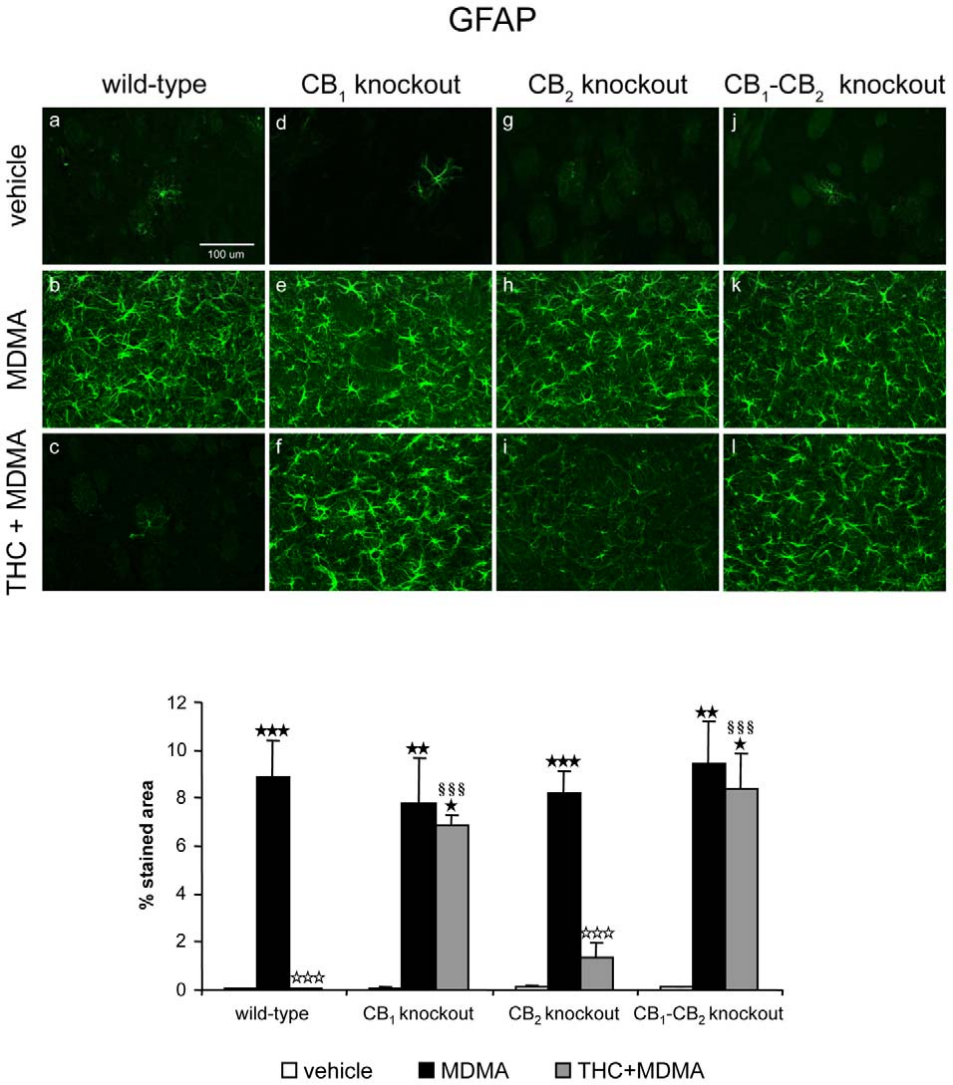
THC prevents MDMA-induced astrocytes activation through CB_1_ and CB_2_ receptor activation. Upper panel: GFAP staining in the striatum of wild-type (a–c), CB_1_ (d–f) CB_2_ (g–i), and double CB_1_/CB_2_ (j–l) knockout mice treated with MDMA (20 mg/kg, i.p.) and THC (3 mg/kg, i.p.), housed at 21±1°C. Animals were sacrificed and brains removed 48 h after the last injection. Scale bar, 100 µm. Lower panel: Data on astrocytes staining are expressed as mean ± SEM of percentage of stained area (*n* = 3–6). ★ *p*<0.05, ★★ *p*<0.01, ★★★ *p*<0.001 when compared with vehicle-treated group. ⋆⋆⋆ *p*<0.001, when compared with MDMA-treated group. §§§ *p*<0.001, when compared with wild-type group (Tukey's test).

The activation of both microglia and astrocytes was evaluated in the striatum of mice housed at room temperature (21±1°C) and treated with THC and MDMA. Staining for activated microglia with an antibody for CD11b (Fig. 2a–c) and for astrocytes with an antibody for GFAP (Fig. 3a–c) showed that MDMA induced a marked glial activation and that THC completely prevented microglia and astrocytes activation induced by MDMA administration. THC alone had no effect on microglia or astrocytes staining (data not shown). To determine the mechanism by which THC prevents microglia and astrocytes activation, CB_1_ (Fig. 2 and 3d–f), CB_2_ (Fig. 2 and 3g–i) and double CB_1_/CB_2_ (Fig. 2 and 3j–l) knockout mice were treated with a THC and MDMA regimen. THC was unable to inhibit microglia and astrocytes activation in CB_1_ and CB_1_–CB_2_ mutant mice (Fig. 2 and 3d–f and j–l). However, THC partially suppressed microglial activation in CB_2_ knockout mice treated with MDMA, and a similar effect was observed with astrocytes activation (Fig. 2 and 3g–i). These results suggest that THC prevented MDMA-induced glial activation by activating CB_1_ receptor, and that the activation of CB_2_ receptor by THC partially contributes to the suppression of MDMA-induced microglial and astrocytes activation (Table 1 and 2).

#### MDMA does not cause visible damage in striatal DA terminals at room temperature

**Figure 4:**
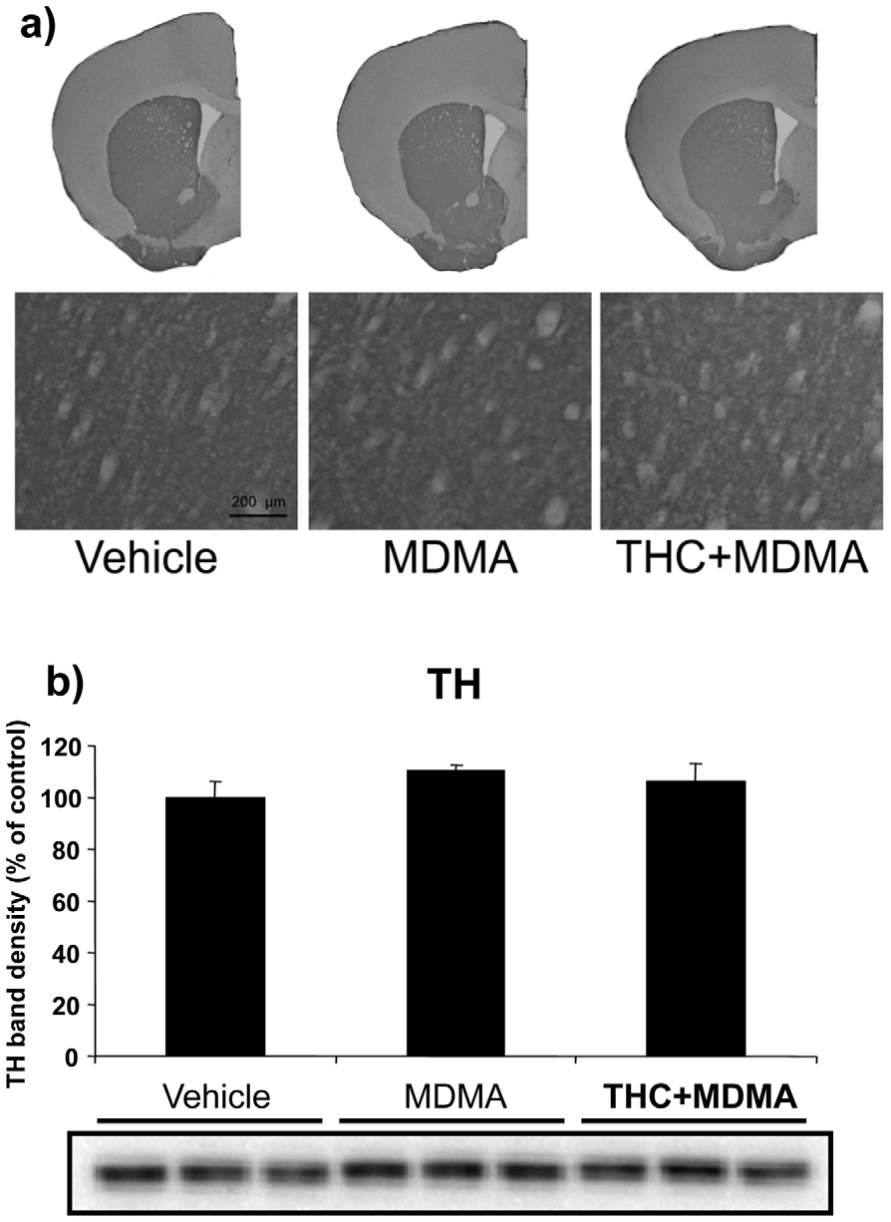
MDMA does not decrease TH levels in the striatum at room temperature. TH immunostaining (a) and protein levels (b) in the striatum of animals treated with THC (3 mg/kg, i.p.) and MDMA (20 mg/kg, i.p.) housed at 21±1°C. Animals were sacrificed and tissue was removed 48 h after the last injection. A representative TH immunostaining (a) is shown. TH band densities (b) were quantified. Data are expressed as mean ± SEM of percentage of density compared to controls (*n* = 3).

To evaluate DA axonal damage, TH levels were evaluated in the striatum of mice treated with THC and MDMA, and housed at 21±1°C (Fig. 4). The structure of the striatum (Fig. 4a) and the content of TH (Fig. 4b) were similar between MDMA and saline treated animals (Table 1). These results suggest that MDMA treatment at room temperature does not cause a significant loss of DA terminals. THC alone had no effects on the structure of the striatum or the levels of TH (data not shown).

### Effects of THC on MDMA Treatment at Warm Temperature (26±1°C)

#### THC reverses MDMA induced hyperthermia at warm temperature by a CB1 receptor mechanism

**Figure 5:**
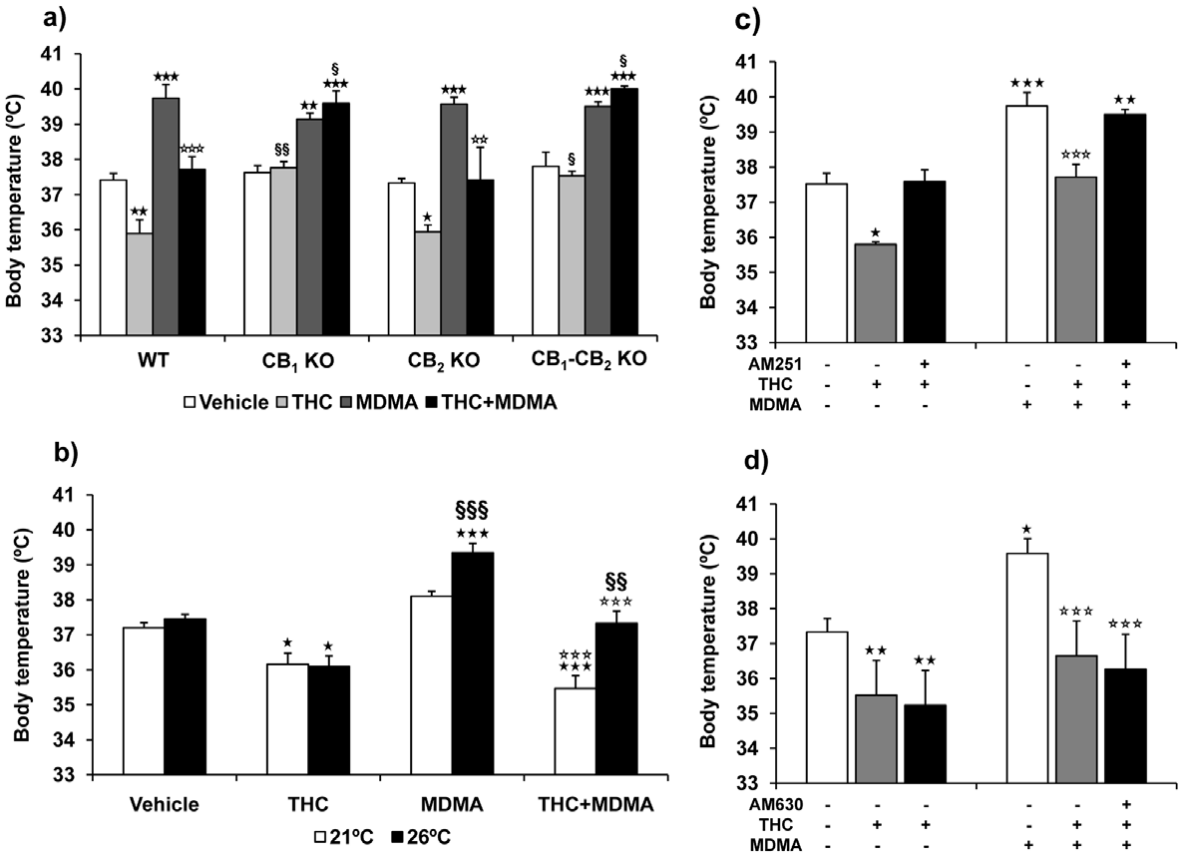
THC prevents MDMA-induced hyperthermia at warm temperature by activating CB_1_ receptor. Effects of pretreatment with THC (3 mg/kg, i.p.) on MDMA (20 mg/kg, i.p.)-induced hyperthermia in (a) wild-type, CB_1_, CB_2_ and double CB_1_/CB_2_ knockout animals housed at 26°±1°C, (b) wild-type animals treated with THC and MDMA at 21 and 26°±1°C, and wild-type animals treated with (c) AM251 or (d) AM630, THC and MDMA at 26°C. Body temperature was measured 30 min after MDMA injection (90 min after THC injection). Data are expressed as mean ± SEM of the body temperature increase (*n* = 6–7). All values are expressed as mean ± SEM. ★★ *p*<0.01, ★★★ *p*<0.001 when compared with vehicle-treated group. ⋆⋆ *p*<0.01, ⋆⋆⋆ *p*<0.001 when compared with wild-type group. § *p*<0.05, §§ *p*<0.01, §§§ *p*<0.001 when compared with wild-type animals or animals housed at 21±1°C (Tukey's test or one-way ANOVA).

Body temperature was measured in animals housed at 26±1°C. These animals showed a baseline core temperature of 37.8±0.05°C, which was similar to basal body temperature of animals housed at 21°C (37.5±0.08°C). This result suggests that increased ambient temperature does not affect basal body temperature. After baseline recordings, wild-type animals were injected with THC followed by MDMA 1 h later, and body temperature was measured 30 min after the MDMA injection (Fig. 5a).

MDMA administration at 26°C produced a significant hyperthermia when compared to the vehicle-treated group (p<0.001), whereas THC alone produced significant hypothermia when compared with control animals (p<0.01). However, body temperature in MDMA-treated animals receiving THC was significantly lower than in animals treated with MDMA alone (p<0.01) and similar to animals treated with vehicle. Thus, THC significantly reduced MDMA-induced hyperthermia at warm temperature (Table 2).

Body temperature of animals housed at 21°C and 26°C were compared (Fig. 5b). No significant differences in core temperature were observed between animals housed at 21°C and at 26°C and treated with vehicle or THC. However, MDMA-treated animals showed a significant enhancement in body temperature when housed at 26°C (p<0.001), and this effect was also observed in MDMA-treated animals receiving THC (p<0.01) (Table 1 and 2). These results indicate that warm ambient temperature potentiates the hyperthermic effects of MDMA, but does not affect hypothermic effects of THC.

Body temperature was also measured in animals pretreated with the CB_1_ receptor antagonist AM251 (Fig. 5c) and the CB_2_ receptor antagonist AM630 (Fig. 5d) receiving THC and MDMA and housed at 26±1°C. AM251 blocked the effect of THC on body temperature. Therefore, THC did not prevent MDMA-induced hyperthermia in these mice. Conversely, THC induced hypothermia and prevented MDMA-induced hyperthermia in animals pretreated with the CB_2_ antagonist AM630 (Table 1).

Body temperature was also recorded in CB_1_, CB_2_ and double CB_1_/CB_2_ knockout mice administered with THC and MDMA (Fig. 5a). Two-way ANOVA (Table 2), and subsequent *post hoc* (Tukey's test) analysis revealed that vehicle-treated CB_1_, CB_2_ and double CB_1_/CB_2_ knockout mice showed similar body temperature than wild-type mice. Moreover, MDMA induced similar hyperthermia in CB_1_, CB_2_ and CB_1_/CB_2_ knockout mice than in wild-type mice. On the contrary, THC induced hypothermia in wild-type and CB_2_ knockout mice, but not in CB_1_ and CB_1_–CB_2_ deficient mice. Consequently, while THC prevented MDMA-induced hyperthermia in wild-type and CB_2_ knockout mice, it was unable to prevent MDMA-induced hyperthermia in mice deficient in the CB_1_ and CB_1_/CB_2_ receptor. Thus, THC prevents MDMA-induced hyperthermia through the activation of CB_1_ receptor.

#### THC prevents microglia and astrocytes activation in MDMA-treated mice housed at warm temperature

**Figure 6:**
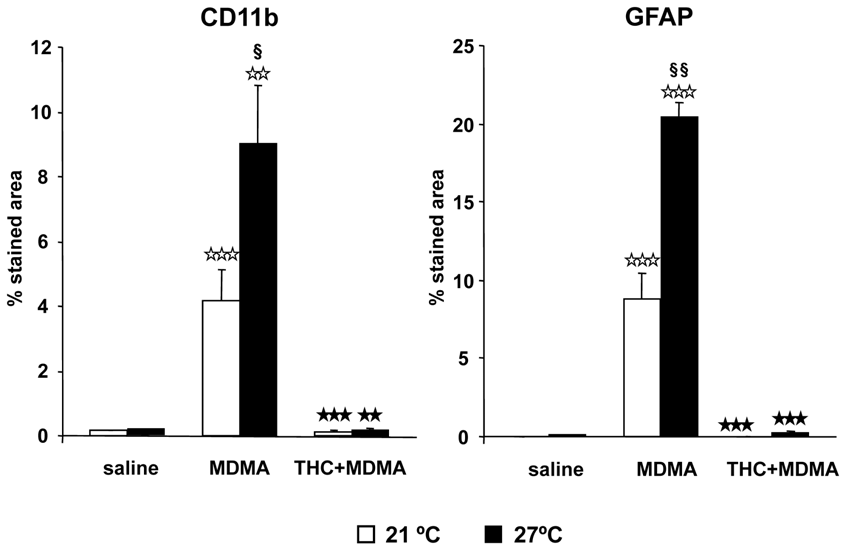
THC prevents MDMA-induced glial activation at both room and warm temperature. CD11b (a) and GFAP (b) staining quantification in mice housed at 21 and 26±1°C. Data are expressed as mean ± SEM of percentage of stained area (*n* = 3–7). ★★★ *p*<0.001 when compared with vehicle-treated group. ⋆⋆⋆ *p*<0.001 when compared with MDMA-treated group. § *p*<0.05, §§ *p*<0.01, when compared with animals housed at 21±1°C (Tukey's test or one-way ANOVA).

Microglia and astrocytes activation was evaluated in the striatum of animals housed at 26±1°C after treatment with THC and MDMA (Figure 6). Microglia (Fig. 6a) and astrocytes (Fig. 6b) were significantly activated in MDMA-treated animals housed at 26°C. However, THC suppressed MDMA-induced microglia and astrocytes activation in animals housed at warm temperature. After, MDMA-induced microglia and astrocytes activation of animals housed at 26°C was compared with animals housed at 21°C. Although the intensity of glial activation was similar at 21 and at 26°C, the area stained with CD11b and astrocytes activation was significantly wider in animals housed at 26°C (Table 2).

#### THC protects against DA terminal loss induced by MDMA at warm ambient temperature by CB1 receptor activation

**Figure 7:**
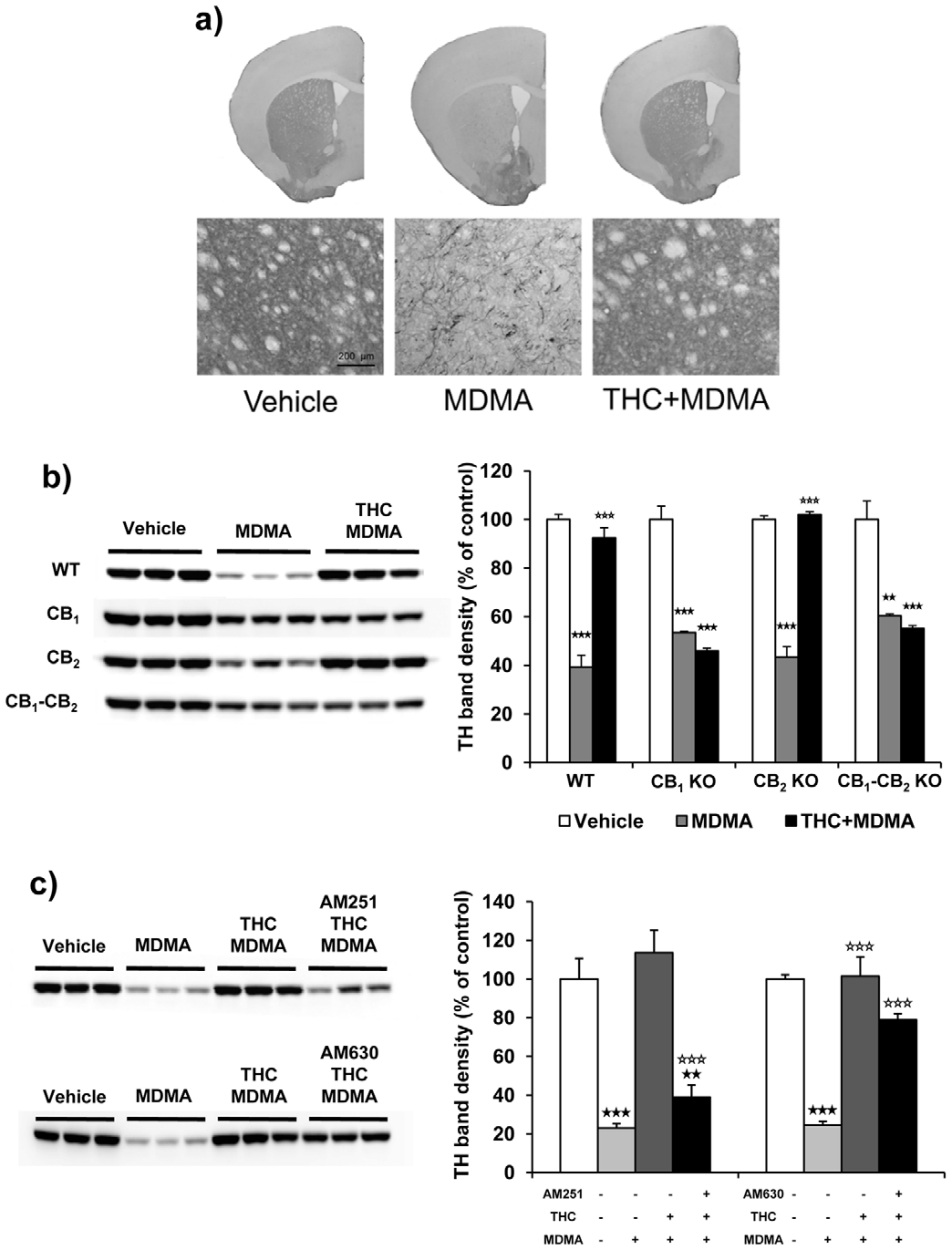
THC prevents MDMA-induced reduction of TH levels in the striatum through CB_1_ activation. Striatum TH immunostaining of wild-type animals treated with THC (3 mg/kg, i.p.) and MDMA (20 mg/kg, i.p.) housed at 26±1°C. A representative TH immunostaining of the whole brain, and 10x images are shown (a). TH protein levels of wild-type, CB_1,_ CB_2_, and double CB_1_/CB_2_ knockout mice (b) and AM251 and AM630-treated mice (c), receiving THC (3 mg/kg, i.p.) and MDMA (20 mg/kg, i.p.) and housed at 26±1°C. A representative TH immunoblot from striatum homogenates is shown. Animals were sacrificed and tissue was removed 48 h after the last injection. TH band densities were quantified. Data are expressed as mean ± SEM of percentage of density compared to controls (*n* = 3). ★★ *p*<0.01, ★★★ *p*<0.001 when compared with vehicle-treated group. ⋆ *p*<0.05, ⋆⋆⋆ *p*<0.001 when compared with MDMA-treated group. § *p*<0.05, §§ *p*<0.01, when compared with wild-type group (Tukey's test).

The integrity of DA terminals was evaluated in animals housed at 26±1°C and treated with THC and MDMA (Figure 7). Unlike animals housed at 21±1°C, the structure of the striatum was notably altered in mice treated with MDMA (Fig 7a), while the striatum integrity of MDMA-treated animals pretreated with THC was similar to the striatum of vehicle-treated animals. Moreover, TH levels of MDMA-treated animals housed at 26±1°C were significantly reduced when compared to vehicle treated animals. In contrast, MDMA-treated mice receiving THC showed similar levels of TH than control animals (Fig. 7b). These results indicate that the administration of MDMA at warm ambient temperature strongly exacerbates DA terminals loss, and that THC attenuates DA terminals damage and loss induced by MDMA in the striatum of animals housed at warm temperature. No differences in TrH or SERT levels were observed in the striatum, prefrontal cortex or hippocampus of animals treated with MDMA (data not shown). This result indicates that MDMA specifically damage DA but not 5-HT terminals in mice.

TH levels were also measured in animals pretreated with the CB_1_ receptor antagonist AM251 and the CB_2_ receptor antagonist AM630 receiving THC and MDMA and housed at 26±1°C (Fig. 7c). THC did not prevent MDMA-induced hyperthermia in mice treated with AM251. Conversely, AM630 did not modify the ability of THC to prevent MDMA-induced hyperthermia (Table 2).

Finally, TH levels were evaluated in CB_1_, CB_2_ and double CB_1_/CB_2_ knockout mice administered with THC and MDMA (Fig. 7b). Vehicle-treated CB_1_, CB_2_ and double CB_1_–CB_2_ knockout mice showed similar TH levels than wild-type mice. In contrast, THC prevented MDMA-induced hyperthermia in wild-type and CB_2_ knockout mice, but it was unable to prevent MDMA-induced DA terminal loss in mice deficient in the CB_1_ and CB_1_–CB_2_ receptor. In conclusion, THC protects against MDMA-induced DA axonal degeneration through the activation of CB_1_ receptor.

## Discussion

In this study, we show that THC prevents MDMA neurotoxicity not only at room temperature, in which brain damage is mild, but also at warm temperature, where the neurotoxic effects of MDMA are strongly enhanced [7], [8]. Many reports describe that MDMA-induced neurotoxicity is directly related to its hyperthermic effect, and that MDMA-induced hyperthermia is proportional to the environmental temperature [8]. For that reason, we tested the hypothermic and neuroprotective effects of THC on MDMA neurotoxicity at both 21°C (room temperature) and 26°C (warm temperature). The hypothermic effects of THC are well known [15], suggesting that THC might be a good candidate to prevent MDMA-induced hyperthermia and neurotoxicity. Furthermore, the anti-inflammatory properties of THC have been widely reported, and cannabinoid drugs have been shown to exhibit strong neuroprotective effects on a wide variety of central nervous system (CNS) disorders [25]. Thus they might contribute to attenuate MDMA neurotoxicity. Additionally, THC seems to have neuroprotective effects mediated by receptor independent mechanisms due to its antioxidant properties [17], [26]. Cannabis, whose main psychoactive compound is THC, is a drug consumed by 95% of MDMA users. The frequent co-use of both drugs makes it particularly interesting to study the effects of their combination. Indeed, previous studies describe the effects of THC and MDMA together in animal models of locomotor activity, temperature, anxiety [18], reward [27] and THC-dependence [19]. However, the neuroprotective effects of THC on MDMA neurotoxicity have never been reported. In addition, the dose of THC used in this study (3 mg/kg, i.p.) could be considered a dose consumed by regular moderate cannabis users, and for that reason similar doses are used in the previously reported animal studies [18], [27].

First, we studied the effects of THC on MDMA-induced hyperthermia. Mice housed at room temperature (21°C) showed a moderated hyperthermia when exposed to MDMA, but severe hyperthermia when room temperature was raised to 26°C. When THC was administered to MDMA-treated animals, hyperthermia was prevented in both, mice housed at 21°C and at 26°C. Consistent with our results, previous studies described that THC attenuated MDMA-mediated hyperthermia in rats [18]. Moreover, unlike MDMA, the effects of THC on body temperature were not affected by ambient temperature. These data indicate that the mechanisms by which THC reduces body temperature are different from those mediating MDMA-induced hyperthermia. While THC specifically activates CB_1_ receptors in the preoptic anterior hypothalamus causing hypothermia [28], MDMA impairs thermoregulation by altering DA and 5-TH systems [29]. To find out the mechanism by which THC prevents MDMA-induced hyperthermia, both THC and MDMA were administered to animals pretreated with the CB_1_ receptor antagonist AM251 or the CB_2_ receptor antagonist AM630, as well as in mice lacking the CB_1_, CB_2_ or both cannabinoid receptors. THC was unable to prevent MDMA-induced hyperthermia in mice treated with AM251 or in mice deficient in the CB_1_ receptor. These data are consistent with other studies that described the unability of THC to avoid MDMA-induced hyperthermia in animals pretreated with the CB_1_ antagonist rimonabant. On the contrary, neither the administration of AM630 nor the deletion of CB_2_ receptor gene modified the ability of THC to prevent MDMA-induced hyperthermia. These results suggest that the hypothermic effects of THC, which are mediated by the activation of CB_1_ receptor [30], are responsible for preventing MDMA-induced hyperthermia, and that CB_2_ receptor has not a predominant role on this effect. Furthermore, there is evidence that the absence of CB_1_ receptor also participates in the expression of MDMA induced hyperthermia [31], indicating that not only the pharmacological activation of this receptor, but its physiological function plays an important role in the hyperthermic effects of MDMA.

Usually, MDMA-induced hyperthermia is related to brain damage. Astrocytes and microglia are activated with minimal alterations in the CNS, and are considered sensitive markers of brain damage. For that reason, we evaluated the effects of THC on MDMA-induced glial activation. MDMA induced a strong activation of microglia and astrocytes in animal housed at both 21 and 26°C, as previously reported [9], [10]. Nevertheless, the administration of THC significantly reduced glial activation in animals housed at both 21 and 26°C. These results agree with previous studies describing that the decrease of MDMA-induced hyperthermia also attenuates glial activation [9], [32]. However, the well known anti-inflammatory properties of THC may also contribute to the reduction of MDMA-induced glial activation. To find out if THC inhibited MDMA-induced microglial and astrocytes activation by preventing hyperthermia or by reducing inflammation, glial activation was evaluated in CB_1_, CB_2_ and double CB_1_/CB_2._ THC was unable to prevent glial activation in CB_1_ and double CB_1_/CB_2_ receptor deficient mice. CB_1_ receptor is necessary to induce the hypothermic properties of THC, which would prevent MDMA hyperthermia and, consequently, cell damage. Furthermore, THC only partially suppressed MDMA-induced astrocytes and microglia activation in CB_2_ knockout mice. Microglia cells express CB_2_ cannabinoid receptor [33], which mediated the anti-inflammatory properties of THC [34]. In many cases, severe microglia activation exacerbates inflammation and brain damage instead of reducing it due to the high amount of inflammatory mediators, such as cytokines and nitric oxide, released by these cells [35]. Thus, the activation of CB_2_ receptor by THC contributes to attenuate microglial activation and, as a result, to reduce cell damage. Thus, both CB_1_ and CB_2_ receptors mediate the THC-induced prevention of MDMA-induced glial activation and cell damage. Furthermore, the receptor-independent antioxidant properties of THC may also contribute to prevent brain damage [17], [26]. However, mice deficient in both CB_1_ and CB_2_ receptors treated with THC and MDMA show the same glial activation than animals treated with MDMA alone. This result indicates that antioxidant properties of THC do not contribute to reduce MDMA neurotoxic effects.

To evaluate if the MDMA-induced brain damage that triggered glial activation resulted in DA axonal loss, we assessed the integrity of DA striatum terminals. DA axons were stained and TH levels were measured in the striatum of mice treated with THC and MDMA and housed at 21 or 26°C. Surprisingly, mice treated with MDMA at 21°C did not show a significant alteration of striatum structure or decrease in TH levels. This result indicates that the cell damage and the consequent inflammation induced by MDMA in animals housed at 21°C were not strong enough to destroy DA nerve terminals. In contrast, MDMA-treated animals housed at 26°C showed an important alteration of striatum structure and a significant decrease of TH levels. These results agree with previous reports describing that the enhancement in MDMA-induced hyperthermia caused by warm ambient temperature also enhances MDMA neurotoxicity [8]. Strikingly, MDMA induced a strong microglia and astrocytes activation at both 21 and 26°C ambient temperatures. Microglia and astrocytes are extremely sensitive to even small pathological changes in the CNS [36], whereas a strong change is required to observe significant changes in striatum structure and TH levels. Thus, the moderated hyperthermia induced by MDMA to animals housed at 21°C caused mild neuronal damage, which elicited an inflammatory reaction, but did not destroy DA axons or reduce TH levels. On the contrary, the strong hyperthermia induced by MDMA at 26°C caused severe neuronal damage that triggered a marked glial activation, an important alteration of the striatum structure, and a significant reduction of TH levels. Our results are supported by other studies showing that warm ambient temperature enhances MDMA neurotoxicity by promoting the formation of neurotoxic derivatives [7], and by enhancing the uptake of these metabolites inside the cell [8], [37]. Hence, the reduction of body temperature by THC may attenuate the formation and uptake of toxic metabolites and prevent MDMA neurotoxicity. Therefore, we evaluated the effect of THC on MDMA-induced DA axonal loss in the striatum of animals housed at 26°C. The striatum of animals treated with THC and MDMA showed similar structure and staining intensity compared with the striatum of saline-treated animals, whereas the striatum of MDMA-treated mice showed a decreased staining and an altered structure. In addition, TH levels in the striatum were similar between saline-treated animals and animals treated with both THC and MDMA, whereas they were strongly reduced in animals treated with MDMA. Thus, THC prevents the destruction of DA axons induced by MDMA at high ambient temperature. To find out the mechanism by which THC prevents DA axonal loss, animals treated with THC and MDMA were pretreated with the CB_1_ antagonist AM251 and the CB_2_ antagonist AM630. THC prevented MDMA-induced DA terminal loss in mice pretreated with AM630 but not with AM251. These results indicate that the activation of CB_1_ but not CB_2_ receptor is necessary for THC to prevent MDMA-induced DA axons destruction. Nevertheless, acute pharmacological blockade of cannabinoid receptors does not inhibit cannabinoid receptor permanently during all the process and axonal degeneration. For that reason, the mechanism by which THC prevents MDMA-induced axonal damage was also evaluated in CB_1,_ CB_2_ and double CB_1_/CB_2_ knockout mice. CB_1_ and double CB_1_/CB_2_ knockout mice treated with THC and MDMA showed similar TH levels than mice treated with MDMA alone, suggesting that the hypothermic effects of THC mediated by CB_1_ receptor are responsible for preventing DA terminals destruction. However, no difference between CB_1_ knockout mice and double CB_1_-CB_2_ knockout treated with both THC and MDMA was observed. Furthermore, no difference between CB_2_ knockout mice and wild-type mice receiving THC and MDMA was observed. Additionally, we observed difference between CB_1_ knockout mice treated with THC and MDMA together and MDMA alone. Altogether, these results indicate that the anti-inflammatory properties of THC mediated by CB_2_ receptor do not significantly contribute to prevent DA terminal loss.

In summary, THC completely prevents MDMA-induced hyperthermia, glial activation and DA axonal loss by inducing CB_1_ receptor-mediated hypothermia. THC anti-inflammatory properties mediated by CB_2_ receptors also reduced microglia and astrocytes activation induced by MDMA. However, THC receptor-independent antioxidant properties do not seem to contribute to reduce MDMA neurotoxicity. Although it has been previously reported that other drugs reducing MDMA-induced hyperthermia also reduced MDMA neurotoxicity [9], these drugs are not used by potential MDMA users. On the contrary, 95% of MDMA users also consume cannabis [14], [21] and, consequently, THC. Some clinical studies have shown that the use of cannabis and MDMA usually results in neurocognitive deficits and neuropsychiatric symptoms, especially in long-lasting heavy users [38]–[40]. However, clinical data suggest the possibility that THC may protect against the neurotoxic effects of MDMA, since several neurological symptoms were attenuated in THC and MDMA consumers when compared to pure MDMA users [21], [41], [42]. Indeed, the use of THC to prevent MDMA-induced hyperthermia and neurotoxicity does not seem to be an adequate measure. However, this study elucidates the consequences of drug polyuse which is one of the main concerns in the study of drugs of abuse, especially MDMA.
